# MicroRNA-383 Regulates the Apoptosis of Tumor Cells through Targeting Gadd45g

**DOI:** 10.1371/journal.pone.0110472

**Published:** 2014-11-21

**Authors:** Lei Zhao, Haihui Gu, Jianfeng Chang, Junyu Wu, Daliang Wang, Su Chen, Xiaomei Yang, Baohua Qian

**Affiliations:** 1 Institute of Epigenetics and Cancer Research, Medical Science Building C-315, School of Medicine, Tsinghua University, Beijing 100084, China; 2 Department of Transfusion Medicine, Changhai Hospital, Second Military Medical University, 168 Changhai Road, Shanghai 200433, China; 3 Research Center for Translational Medicine at East Hospital, School of Life Sciences and Technology, Tongji University, 150 Jimo Road/1239 Siping Road, Shanghai 200120/200092, China; University of Kentucky College of Medicine, United States of America

## Abstract

***Background*:**

MicroRNAs (miRNAs) are a class of small non-coding single-stranded RNA molecules that inhibit gene expression at post-transcriptional level. Gadd45g (growth arrest and DNA-damage-inducible 45 gamma) is a stress-response protein, which has been implicated in several biological processes, including DNA repair, the cell cycle and cell differentiation.

***Results*:**

In this work, we found that miR-383 is a negative regulator of Gadd45g. Forced expression of miR-383 decreased the expression of Gadd45g through binding to the 3′ untranslated region (3′-UTR), whereas inhibition of miR-383 increased Gadd45g expression. The presence of miR-383 increased the cellular sensitivity to DNA damage in breast cancer cells, which was rescued by ectopic expression of Gadd45g without the 3′-UTR. miR-383 also regulates the expression of Gadd45g in embryonic stem (ES) cells, but not their apoptosis under genotoxic stress. miR-383 was further showed to negatively regulate ES cell differentiation via targeting Gadd45g, which subsequently modulates the pluripotency-associated genes. Taken together, our study demonstrates that miR-383 is a negative regulator of Gadd45g in both tumor cells and ES cells, however, has distinct function in regulating cell apoptosis. miR-383 may be used as antineoplastic agents in cancer chemotherapy.

***Conclusion*:**

We demonstrate for the first time that miR-383 can specifically regulates the expression of Gadd45g by directly targeting to the 3-UTR region of Gadd45g mRNA, a regulatory process conserved in human tumor cells and mouse embryonic stem cells. These two compotents can be potentially used as antineoplastic agents in cancer chemotherapy.

## Introduction

MicroRNAs (miRNAs) are a class of small non-coding single-stranded RNA molecules that inhibit gene expression at post-transcriptional level [1]. In the cell nucleus, miRNAs are transcribed and cleaved by Drosha and DGCR8 to form precursor miRNAs (pre-miRNAs) [2],[3]. Pre-miRNAs are further exported to the cytoplasm by exportin 5 (XPO5) [4]. In the cytoplasm, pre-miRNAs are processed by Dicer and TAR RNA-binding protein 2 (TARBP2) to produce mature miRNAs (∼22nts), which are finally loaded in the RNA induced silencing complex (RISC) [5]. The miRNA-RISC results in mRNA cleavage or translation repression, through which miRNAs play key roles in various biological processes [6],[7]. It has been reported that the translation of more than 60% of the protein-coding genes are mediated by miRNAs [5]. Defects in miRNAs are known to be a factor in many diseases [8],[9].

Under genotoxic stress such as UV irradiation, DNA is continuously undergoing damage, which in turn elicits cellular responses, including activation of the DNA repair pathway, cell cycle arrest and apoptotic cell death [10]. Multiple miRNAs have been found to be involved in regulating the sensitivity to genotoxic stress. miR-24 was found to increase the sensitivity to genotoxic drugs in differentiated blood cells by down-regulating H2AX [11]. miR-421 induces cells to become hypersensitive to ionizing radiation, which is dependent on ATM [12]. Ectopic expression of miR-214 confers resistance to cisplatin in ovarian cancer cells by targeting PTEN [13]. miR-504 reduces etoposide-caused apoptosis by targeting p53 [14].

Recently, emerging evidence has shown that miRNAs also participate in controlling the fate of embryonic stem cells (ES cells). For example, ES cells lacking Dicer or DGCR8 exhibit defects in differentiation and proliferation [15],[16]. Transcriptional factors such as Sox2, Oct4 and Nanog are important pluripotency genes and play essential roles in self-renewal of ES cells [17]. These genes have been shown to be silenced by various miRNAs, such as miR-134, miR-145, miR-296 and miR-470 [18]. Thus, understanding the roles of miRNAs in ES cells would help elucidate the regulatory network involved in ES cell self-renewal and differentiation.

Gadd45g is a member of Gadd45 family that contains the additional two members, Gadd45a and Gadd45b. They are closely associated with cell growth, DNA repair, cell cycle and apoptosis [19]. These three proteins share approximately 60% identity at the amino acid level and exert their functions through interacting with additional proteins, such as PCNA, p21, cdc2/cyclinB1 and MTK1/MEKK4 [20]–[23]. Gadd45a or Gadd45b deficient mouse hematopoietic cells are more sensitive to UV-induced damage [24],[25]. Gadd45g has been reported to be up-regulated after UV irradiation in both normal and tumor cells [26],[27]. In addition, Gadd45 genes are also reported to be involved in the processes of embryonic development and differentiation in several species [28]–[31]. Recently, Gadd45g was implicated in male sex determination by regulating *Sry* expression [32],[33]. However, little is known about whether miRNAs participate in responding to stress stimulation or cell differentiation through the Gadd45 genes.

In this study, we found that Gadd45g is a direct target of miR-383, and miR-383 is able to increase the sensitivity of breast cancer cells to both UV irradiation and cisplatin treatment. Notably, miR-383 regulates the expression of Gadd45g in ES cells, but not their apoptosis. These findings provide new insights into the mechanism of miRNAs in the regulation of cellular sensitivity to genotoxic drug treatments in breast cancer cells. Moreover, miR-383 is suggested to function as a negative regulator of embryonic stem cell differentiation via down-regulation of Gadd45g expression.

## Results

### miR-383 down-regulates Gadd45g by directly targeting the 3′-UTR of Gadd45g

**Figure 1:**
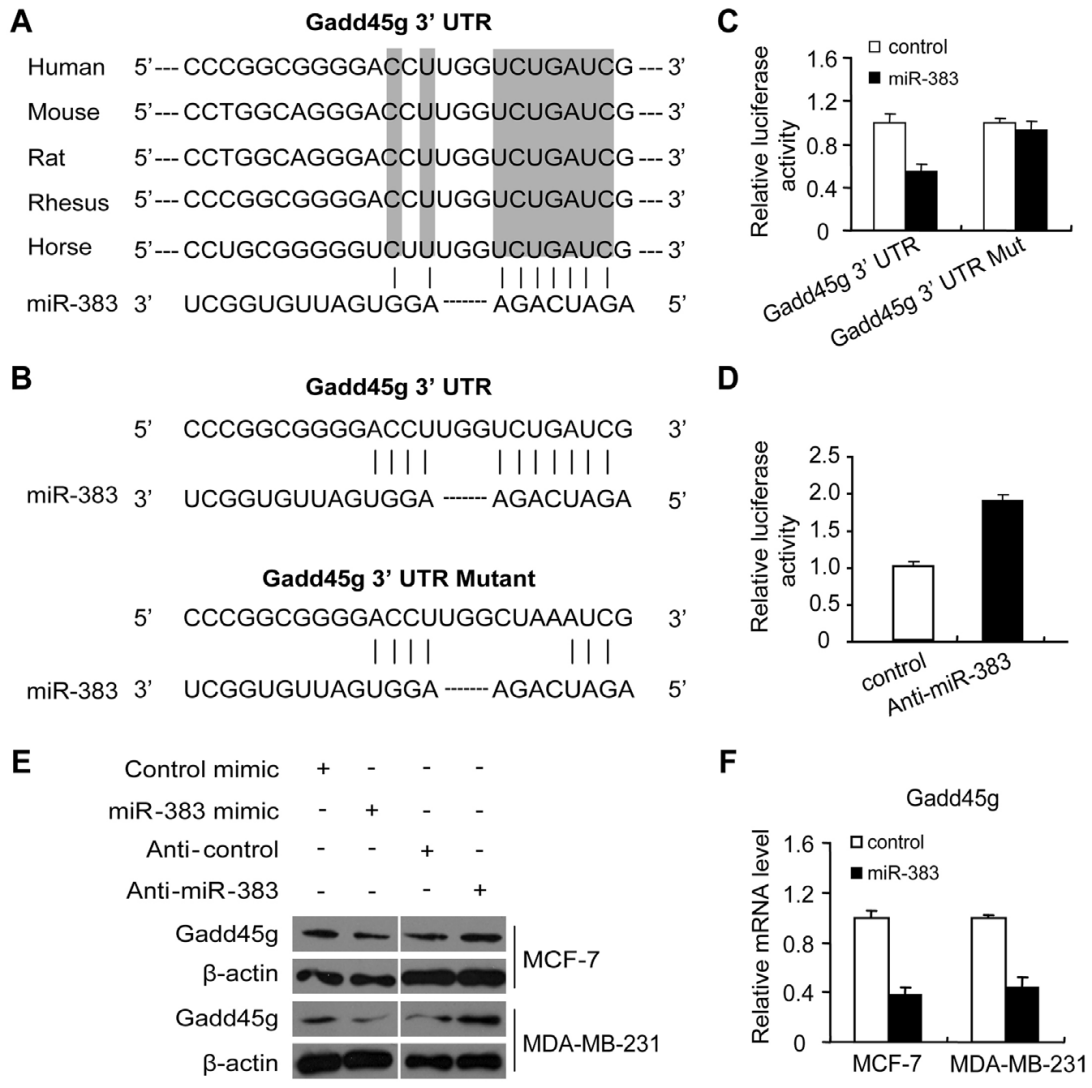
miR-383 represses Gadd45g expression by directly targeting Gadd45g 3′-UTR. (A) Schematic representation of miR-383 binding site on the Gadd45g 3′-UTR. Shaded texts indicate the conserved sequences among human, mouse, rat, rhesus monkey and horse. (B) Gadd45g 3′-UTR sequence containing the predicted target sites was inserted into the pMIR reporter vector, immediately downstream the luciferase gene. The mutant reporter construct was generated by introducing four-mismatch mutation. (C) Relative luciferase activities of Gadd45g 3′-UTR reporter or mutated Gadd45g 3′-UTR reporter in MCF-7 cells with or without miR-383 mimic. Firefly luciferase reading was normalized to that of the Renilla luciferase. Values are means ± SD. (D) MCF-7 cells were co-transfected with the Gadd45g 3′-UTR reporter construct, and anti-miR-383 or anti-control, supplemented by pRL vector, and luciferase activities were analyzed after 48 h. Values are means ± SD. (E) The effect of miR-383 mimic or anti-miR-383 on Gad4d45g protein levels. Protein expression of Gadd45g was determined by western blotting in MCF-7 and MDA-MB-231 cells at 48 h after transfection. β-actin was used as a loading control. (F) Relative Gadd45g mRNA expression was measured by qRT-PCR in MCF-7 and MDA-MB-231 cells transfected with miR-383 mimic or control. Levels were normalized to GAPDH expression. Values are means ± SD.

Given the important roles of Gadd45 in DNA damage repair and cell growth/differentiation, we were interested in examining the upstream regulators of Gadd45g, such as miRNAs. We therefore used three computer-aided algorithms (TargetScan, miRBase and Picta) to search for potential miRNA-binding sites in Gadd45g mRNA. One miRNA, miR-383, was found to target Gadd45g using the three algorithms, and the putative binding site of miR-383 in the 3′-UTR of Gadd45g is highly conserved in different species (human, mouse, rat, rhesus monkey and horse) (Fig. 1A). This suggests that miR-383 is a possible regulator of Gadd45g.

We next used a luciferase reporter assay to validate the binding of miR-383 to the 3′-UTR of Gadd45g. The wild-type Gadd45g-3′-UTR or mutant Gadd45g-3′-UTR were cloned into the pMIR-REPORT luciferase vector downstream from its Firefly luciferase gene (Fig. 1B). The wild-type or mutant pMIR-Gadd45g-3′-UTR reporter was co-transfected with a control or a miR-383 mimic plasmid, and a pRL-SV40 vector containing the Renilla luciferase gene was also co-transfected as a normalization control. The activity of the Firefly luciferase construct containing wild-type 3′-UTR of Gadd45g was suppressed by ectopic expression of miR-383 as compared with control (Fig. 1C). However, this suppression was abolished when the 3′-UTR of Gadd45g was mutated (Fig. 1C). Anti-miR-383 was also used to co-transfected with luciferase construct containing wild-type 3′-UTR of Gadd45g, and the luciferase activity was increased by anti-miR-383 as compared with control (Fig. 1D).

To investigate the regulation of Gadd45g by miR-383 *in vivo*, we examined the protein level of Gadd45g under a condition of overexpression of miR-383 mimic or anti-miR-383. As shown in Fig. 1E, the endogenous protein levels of Gadd45g were reduced by ectopic expression of miR-383 in MCF-7 and MDA-MB-231 cells. Additionally, transfection of MCF-7 or MDA-MB-231 cells with anti-miR-383 increased the Gadd45g protein levels (Fig. 1E). We further observed that endogenous Gadd45g mRNA was also down-regulated by miR-383 mimic in both MCF-7 and MDA-MB-231 cells (Fig. 1F). Taken together, these data indicate that Gadd45g is a direct target of miR-383.

### Gadd45g and miR-383 are negatively correlated in response to UV irradiation

**Figure 2:**
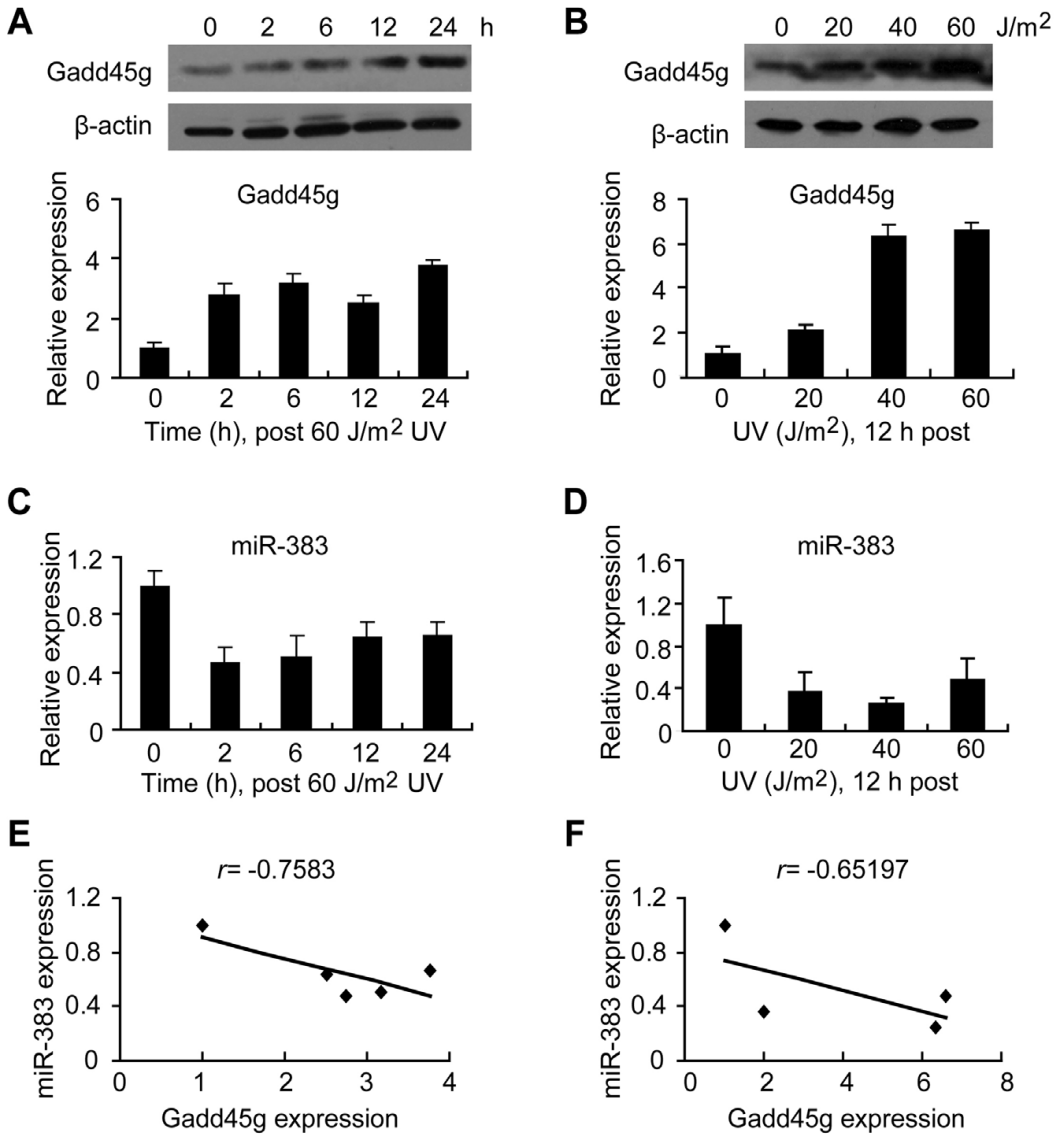
The expression of miR-383 and Gadd45g is negatively correlated post UV irradiation. (A) The mRNA level of Gadd45g was measured by qRT-PCR post UV irradiation (60 J/m^2^) at indicated time in MCF-7 cells, and normalized to those of GAPDH (top). The mRNA level at 0 h was set as 1.0. The protein level of Gadd45g was measured by western blotting, and β-actin was used as a loading control (bottom). (B) Relative Gadd45g mRNA expression was measured by qRT-PCR 12 h post UV irradiation at different doses (top). The data at 0 J/m^2^ was set as 1.0. Western blotting was used to analyze Gadd45g protein levels. β-actin was used as a loading control (bottom). (C and D) The expression of miR-383 was measured by qRT-PCR post UV irradiation (60 J/m^2^) at indicated time (C) or different doses (D) in MCF-7 cells, and normalized to the level of U6. The data at 0 h or 0 J/m^2^ was set as 1.0. Values are means ± SD. (E and F) A significant negative correlation was found between miR-383 and Gadd45g expression under UV irradiation post different time or at different doses.

It has been well known that UV irradiation induces an increased expression of Gadd45g in a variety of cell lines [26],[27]. In order to determine the regulatory relationship between miR-383 and Gadd45g, we examined the expression levels of Gadd45g and miR-383 post UV irradiation. MCF-7 cells were treated with different doses of UV ranging from 20 to 60 J/m^2^ or monitored for up to 24 h after irradiation at 60 J/m^2^. Induction of apoptosis was confirmed by the appearance of condensed chromatin or apoptotic bodies after irradiation in both a time- (Fig. S1A) and dose- (Fig. S1B) dependent manner. After UV irradiation at 60 J/m^2^, the mRNA and protein levels of Gadd45g were elevated in a time-dependent manner (Fig. 2A). In addition, when MCF-7 cells were irradiated by UV at different doses, the Gadd45g expression also exhibited an elevation following the increased UV dose (Fig. 2B). We next examined the expression levels of miR-383 in MCF-7 cells before and after UV irradiation by qRT-PCR. The levels of endogenous miR-383 were reduced in a time and dose dependent manner in response to UV irradiation (Fig. 2C and D). As shown in Fig. 2E and F, statistical analysis showed that Gadd45g mRNA expression levels are inversely correlated with miR-383 (r = −0.7583, r = −0.65197, P<0.05). These results therefore support a role for miR-383 in controlling Gadd45g expression during the cellular response to UV irradiation, and the down-regulation of miR-383 may be involved in UV-induced apoptosis.

### miR-383 regulates the cellular apoptotic sensitivity to UV or cisplatin

To investigate the role of miR-383 in regulating cellular sensitivity to multiple stresses, we examined the effects of miR-383 on the cellular responses to UV irradiation and cisplatin, a widely used chemotherapy drug in cancer patients. After transfection with miR-383 mimic or control, MCF-7 cells were irradiated with UV (60 J/m^2^). At 12 hours after UV irradiation, morphologic examination performed under light microscopy indicated that there were more cells displaying apoptosis in miR-383 transfected cells than control cells (Fig. S2A). Cisplatin is a cytotoxic agent that induces apoptosis through DNA cross-linking. The effect of miR-383 on the response to cisplatin in MCF-7 cells was also examined, and we found that miR-383 overexpressing cells exhibited a more severe cell death than control cells upon cisplatin treatment (Fig. S2A). We also observed similar results in another breast cancer cell line, MDA-MB-231. The miR-383 mimic transfected MDA-MB-231 cells exhibited an increased sensitivity to UV irradiation and cisplatin compared with control cells as well (Fig. S2B).

**Figure 3:**
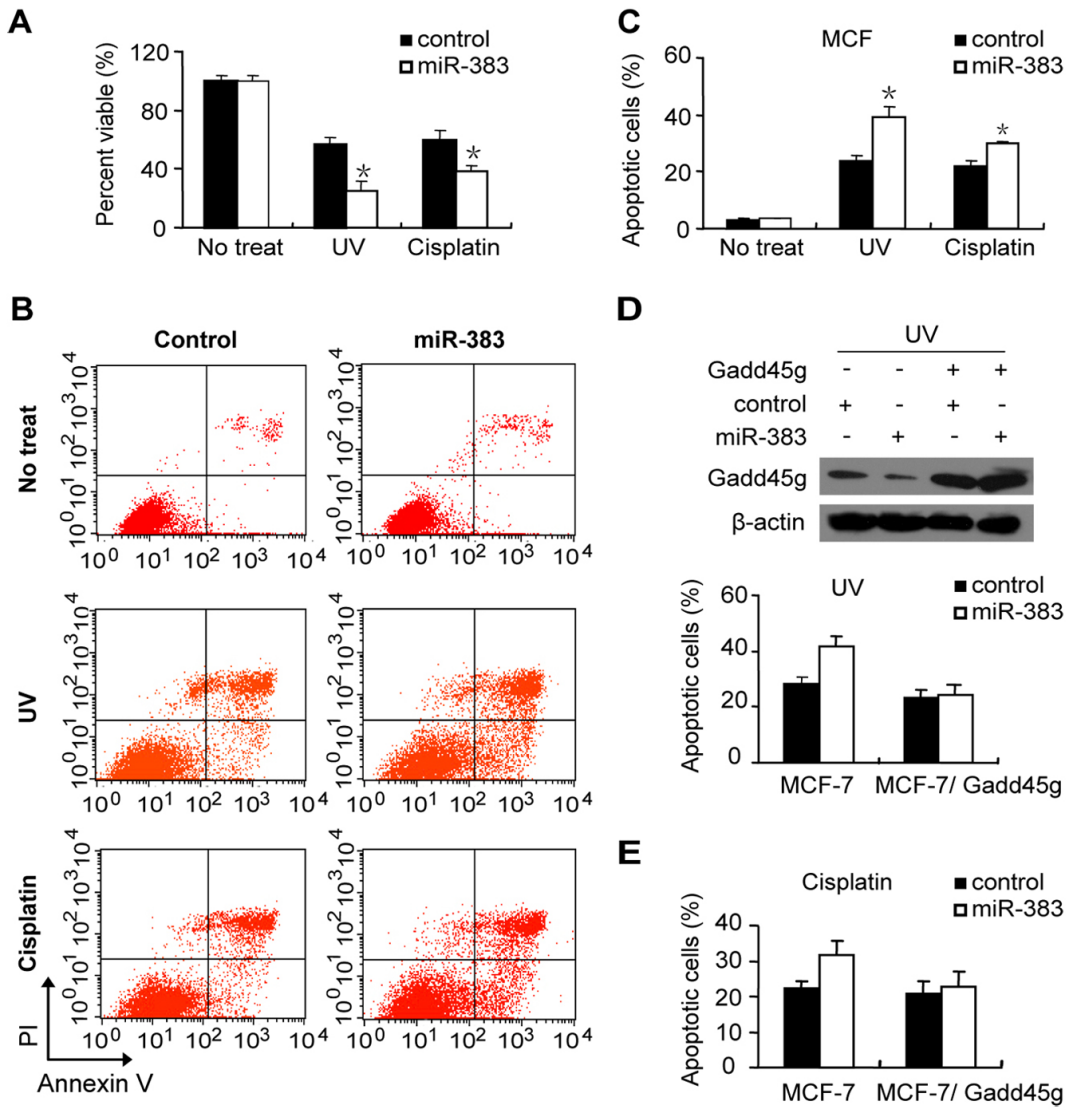
miR-383 regulates cellular apoptotic sensitivity to genotoxic stress through targeting Gadd45g. (A) MTT assays were performed in miR-383 mimic or a control plasmid transfected MCF-7 cells following treatment with UV irradiation (60 J/m^2^, post 12 h) or cisplatin (25 µM, post 24 h). (B) Apoptosis was analyzed by Annexin V/PI assays in MCF-7 cells transfected with miR-383 mimic or control followed by with UV irradiation or cisplatin treatment. Percentage of apoptotic cells was shown at bottom. (C) MCF-7 cells were co-transfected with miR-383 mimic or control and Gadd45g expression plasmid without the 3′-UTR. After transfection, cells were treated by UV irradiation (60 J/m^2^, post 12 h). Gadd45g levels were reduced by miR-383 mimic, and rescued by the Gadd45g expression plasmid (top). Apoptosis was analyzed by Annexin V/PI assay (bottom). (D) MCF-7 cells were co-transfected with miR-383 mimic or control and Gadd45g expression plasmid without the 3′-UTR, and treated with cisplatin (25 µM, post 24 h). Apoptosis was analyzed by Annexin V/PI assay.

MTT assay was also used to assess cell viability, and we found that the viability of miR-383 mimic transfected MCF-7 cells decreased more significantly than control cells after UV irradiation or cisplatin treatment (Fig. 3A). The effect of miR-383 on cell viability was further confirmed in MDA-MB-231 cells treated with UV irradiation or cisplatin. miR-383 enhanced the cytotoxicity caused by both types of genotoxic stress in MDA-MB-231 cells (Fig. S3A).

To confirm the decreased cell viability induced by miR-383 was due to apoptosis, Annexin V/PI assays were performed 12 h following UV irradiation or 24 h following cisplatin treatment. As shown in Fig. 3B, nearly two folds of MCF-7 cells show positive staining with Annexin V in miR-383 transfected cells than in control cells. Similar results were also obtained in MDA-MB-231 cells (Fig. S3B). These results indicate that miR-383 increases the sensitivity of breast cancer cells in response to UV irradiation and cisplatin treatment, and thus enhances stress-induced apoptosis.

### The effect of miR-383 on cell apoptosis caused by genotoxic stress is mediated by Gadd45g

To ascertain the roles of Gadd45g in miR-383 regulated apoptotic sensitivity induced by genotoxic stress, MCF-7 cells were co-transfected with miR-383 construct and a miR-383-insensitive Gadd45g expression plasmid without the 3′-UTR. Fig. 3C shows that Gadd45g expression plasmid rescues the down-regulation of Gadd45g induced by miR-383 (Fig. 3C, upper panel). Apoptosis assay shows that the effect of miR-383 on cell apoptosis induced by UV irradiation was fully rescued by overexpressing Gadd45g lacking the 3′-UTR in MCF-7 cells (Fig. 3C, lower panel). Similarly, the apoptosis after cisplatin treatment is increased in miR-383 mimic transfected cells, but this increase is only happened in the absence of miR-383-resistent Gadd45g plasmid (Fig. 3D). Taken together, these data demonstrate that inhibition of Gadd45g expression is at least partially responsible for the miR-383 enhanced apoptotic sensitivity to genotoxic stress.

### Exctopic expression of miR-383 does not affect apoptosis in ES cells

**Figure 4:**
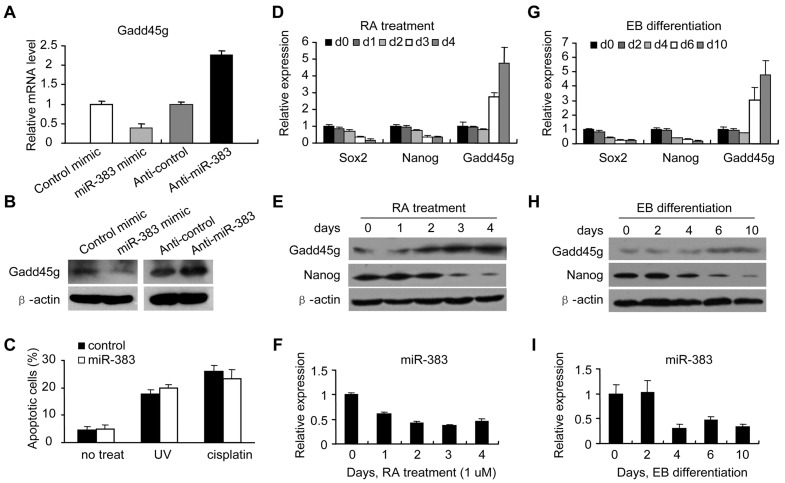
miR-383 regulates Gadd45g in mouse ES cells. (A) Relative mRNA expression of Gadd45g was measured by qRT-PCR in mouse ES cells (R1) transfected with miR-383 mimic or anti-miR-383. (B) Gadd45g protein levels were determined by western blotting in R1 cells. β-actin was used as a loading control. (C) R1 cells were transfected with miR-383 or control, followed by treatment with UV irradiation (50 J/m^2^, 12 h) or cisplatin treatment (10 µM, 24 h). The apoptosis was measured by Annexin V/PI staining. (D) Quantitative RT-PCR and (E) western blotting analysis were performed in ES cells and differentiated ES cells treated with RA (1 µM) for indicated days. (F) Quantitative RT-PCR data for miR-383 were performed in cells mentioned in (D), and normalized to the level of U6. (G) Quantitative RT-PCR and (H) western blotting analysis were performed in ES cells and cultured EB. (I) Quantitative RT-PCR data for miR-383 were performed in ES cells and cultured EB. In (D) and (G), data are expressed as relative expression compared with ES cells (set as 1.0). Values are means ± SD. In (E) and (H), β-actin was used as a loading control.

To determine whether the relationship between miR-383 and Gadd45g are conserved in physiological conditions, we employed mouse ES cell as model system. We found that ectopic expression of miR-383 in R1 ES cells also results in the down-regulation of Gadd45g both at the mRNA and protein levels, while anti-miR-383 up-regulates Gadd45g (Fig. 4A and B), suggesting a consistent role of miR-383 in regulation Gadd45g. However, miR-383 overexpression has almost no effect on cell apoptosis after UV irradiation or cisplatin treatment in ES cells (Fig. 4C), suggesting distinct function of miR-383 in the response to DNA damage between ES cells and tumor cells [34].

### miR-383 negatively modulates ES cell differentiation through Gadd45g

The expression pattern of miR383 and Gadd45g was further studied during ES cell differentiation. The mouse ES cell line R1 was treated with with *Rotinoid Acid* (RA) for differentiation, and we found that miR-383 expression was down-regulated in during ES cell differentiation (Fig. 4F). In contrast, Gadd45g was up-regulated at both mRNA and protein levels (Fig. 4D and 4E). The inversed correlation between Gadd45g and miR-383 was also observed in spontaneous differentiation of embryonic body (EB). Fig. 4G, H and I showed that miR-383 was decreased in parallel with the increase of Gadd45g expression. These results raise a possibility that miR-383 regulates Gadd45g in the process of ES cell differentiation.

**Figure 5:**
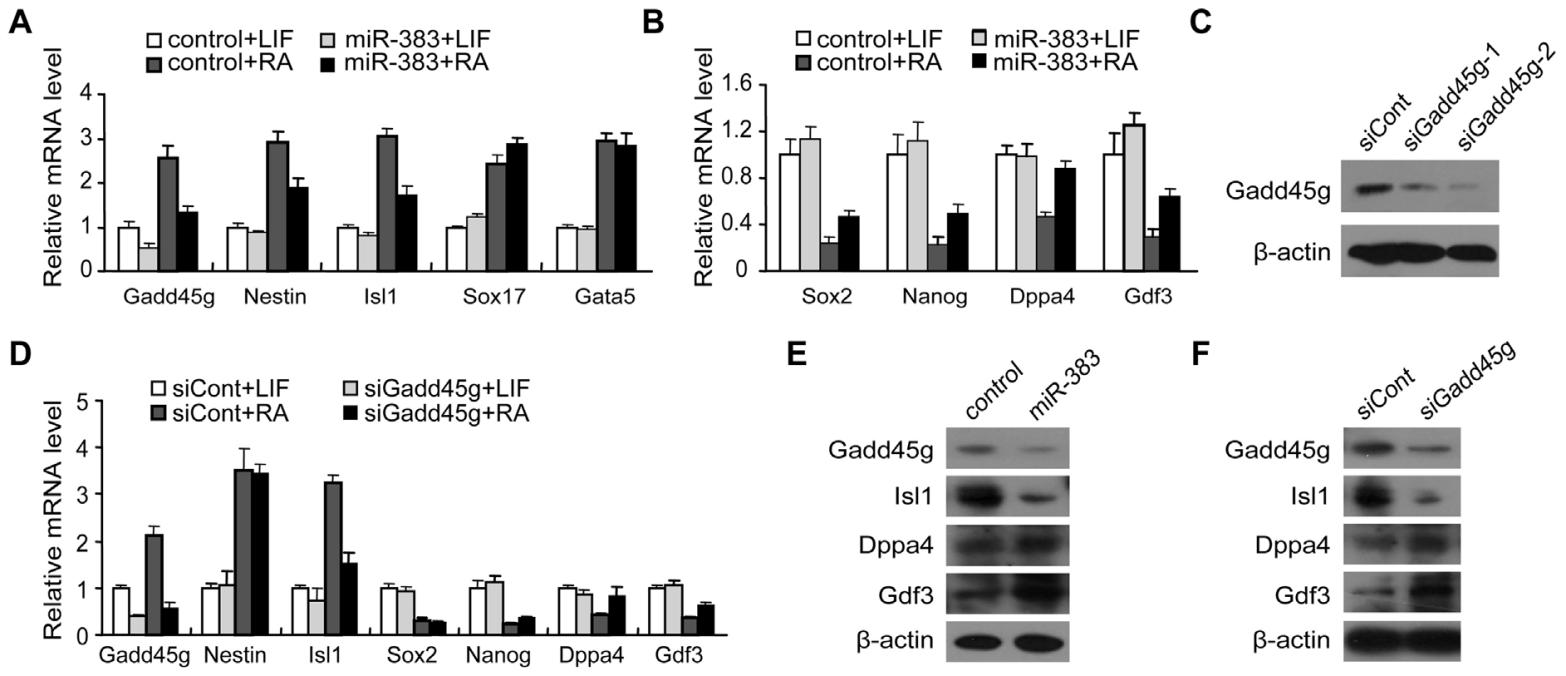
miR-383 modulates ES cell differentiation through Gadd45g. (A and B) Quantitative RT-PCR analysis for differentiation (A) and pluripotency (B) marker genes in miR-383 mimic or control transfected ES cells cultured with LIF or with RA for 3 days. The data are shown as relative expression compared with the control cells cultured in the presence of LIF (set as 1.0). Values are means ± SD. (C) Protein levels of Gadd45g were detected in R1 cells transfected with Gadd45g siRNAs. (D) Quantitative RT-PCR was performed to analyze the expression of Nestin, Nanog, Sox2, Dppa4, Gdf3, and Isl1 between control and Gadd45g siRNA transfected ES cells cultured with LIF or RA. The mRNA levels at control siRNA transfected cells were set at 1.0. Values are means ± SD. (E and F) The protein levels of Isl1, Dppa4 and Gdf3 were examined by western blotting after miR-383 overexpression (E)or Gadd45g siRNA (F) in the conditions of RA treatment.

To further evaluate the role of miR-383 in ES cell differentiation, we overexpressed miR-383 mimic in ES cells followed by RA treatment for 3 days. An increased expression of Gadd45g and the differentiation markers, Nestin and Isl1 (Fig. 5A), and a decreased expression of the pluripotency markers, Sox2, Nanog, Dppa4 and Gdf3 (Fig. 5B), was observed in control cells during RA induction by qRT-PCR. However, the RA-induced up-regulation of Gadd45g was inhibited by miR-383 in miR-383 overexpressed R1 cells (Fig. 5A). Moreover, following RA treatment, up-regulation of Nestin and Isl1, and down-regulation of Sox2, Nanog, Dppa4 and Gdf3 were blocked by overexpression of miR-383 compared with those in control cells (Fig. 5A and B). Thus, miR-383 has the potential to inhibit ES cell differentiation by regulating these genes. We also compared the expression of other differentiation associated genes, such as Sox17 and Gata5, but miR-383 had no effect on the expression of these genes (Fig. 5A).

Dppa4 and Gdf3 are pluripotency-associated genes that are required for maintaining the undifferentiated state of ES cells [35],[36]. Isl1 is a marker of neuron differentiation, playing a significant role in the development of motor neurons and interneurons [37],[38]. These three genes have been observed to be regulated by Gadd45g in hEC cells [39]. The above results suggest that these genes are likely to be regulated by miR-383-Gadd45g axis. To address the possibility, we next tested the effect of Gadd45g on the expressional levels of the selected pluripotency and differentiation related genes during ES cell differentiation. We depleted Gadd45g expression by RNAi. As shown in Fig. 5C, two Gadd45g siRNAs were evaluated and siRNA-2 was found to be more efficient in decreasing Gadd45g expression and was thus used for following experiments. In RA-induced ES cells, when Gadd45g was knocked down, the increase of Isl1 expression and the decrease of Dppa4 and Gdf3 expression in response to ES cell differentiation were inhibited, which was comparable to the effect of miR-383 overexpression (Fig. 5D). The expression of Sox2, Nanog and Nestin was not changed by Gadd45g RNAi (Fig. 5D). The protein levels of Isl1, Dppa4 and Gdf3 were also examined after Gadd45g depletion or miR-383 overexpression. Both miR-383 overexpression (Fig. 5E) and Gadd45g depletion (Fig. 5F) down-regulated Isl1 protein levels and up-regulated Gdf3 and Dppa4 protein levels. These results support that miR-383 functions as a negative regulator of ES cell differentiation by targeting Gadd45g.

## Discussion

UV irradiation of mammalian cells is known to have apoptotic effects and cisplatin is a commonly used chemotherapeutic agent that activates cellular apoptosis via DNA cross-linking [40]. In cancer treatment, the process of apoptosis plays a critical role in the removal of damaged cells [41]. Therefore, agents that can induce genotoxic and metabolic stress are widely used in clinical cancer therapy. One problem is that tumor cells are commonly capable of developing resistance to these treatments. In order to overcome this, it is necessary to increase the sensitivity of tumor cells to these genotoxic agents. In our studies, we found that the expression of miR-383 is involved in cellular response to genotoxic stress. The expression profiles of miRNAs are altered in response to various events. For example, treatment with ionizing radiation, H_2_O_2_, etoposide or UV irradiation can induce an alteration of the miRNA expression profile [42],[43]. The miRNA profiling was carried out in primary human fibroblasts at a low dose of UV irradiation, but miR-383 was found to be unchanged [43]. This may be due to the different cell lines, dosages and time points used [44]. Here, we report that overexpression of miR-383 in breast cancer cells increased the apoptotic sensitivity to UV or cisplatin, indicating that miR-383 regulates cell apoptosis induced by genotoxic stress. Intrerestingly, miR-383 was not involved in the apoptosis of ES cells under the same genotoxic stress. Therefore, miRNAs, including miR-383, are candidate antineoplastic agents based on their capacity to alter the responsiveness to cytotoxic agents [45].

miRNAs regulate gene expression by binding mRNA, thereby resulting in mRNA degradation or protein translation inhibition. In our experiments, we demonstrated that miR-383 binds to the 3′-UTR of Gadd45g so as to down-regulate its expression. Gadd45g, as well as Gadd45a and Gadd45b, respond to environmental stress by mediating activation of the p38/JNK pathway via MTK1/MEKK4 kinase [23]. In our studies, we determined that the expression of Gadd45g was elevated post UV irradiation, which result is inversely correlated with miR-383 expression. This suggests that miR-383 is likely the upstream modulator of Gadd45g in response to environmental stress. Further, when Gadd45g cDNA lacking the 3′-UTR was overexpressed in breast cancer cells, the enhancement of cytotoxic agents-induced apoptosis by miR-383 was rescued indicating that the key target of miR-383 in regulating the sensitivity to genotoxic stress is Gadd45g. A previous report has shown that in testicular embryonic carcinoma cells miR-383 induces a decreased proliferation rate by targeting interferon regulatory factor-1 (IRF1) [46], implying that miR-383 exerts effects through various targets.

Moreover, we showed that Gadd45g is also a target of miR-383 in mouse ES cells. Both the mRNA and protein levels of Gadd45g were regulated by miR-383. In addition to affecting cell death and cell cycle, mouse Gadd45g was shown to be up-regulated during embryonic development and highly expressed in neurons [28]–[30]. Gadd45g has also been shown to promote neuronal differentiation in Xenopus and hEC cells. The knockdown of both Gadd45a and Gadd45g in Xenopus decreases neural crest markers and increases multipotency markers [31]. Gadd45g overexpression in hEC cells elevates neuronal markers, while it down-regulates pluripotency associated genes [39],[47]. Additionally, miR-383 has been shown to be down-regulated in mouse testis after birth until 14 days postpartum [46], suggesting that miR-383 may be involved in mouse embryonic development. Consistent with these data, in our experiments, miR-383 is down-regulated during RA-induced ES cell differentiation or ES spontaneous differentiation, coupled with an up-regulation of Gadd45g. Thus, we assume that miR-383 participates in regulating ES cell differentiation through targeting Gadd45g.

We further observed that, in RA-induced differentiating ES cells, an overexpression of miR-383 or depletion of Gadd45g suppressed the expression of Isl1 and increased the expression of Dppa4 and Gdf3. Although the function of Dppa4 and Gdf3 is not as well understood as Nanog, Sox2 and Oct4, most studies suggest their involvement in ES cell self-renewal and pluripotency. Dppa4 down-regulated ES cells are hard to maintain in the undifferentiated state [48] and Gdf3 is specifically expressed in ES cells [36]. Isl1 is an important gene in development of islets, neurons and cardiac tissue [49]. Our results suggest that miR-383 regulates these ES cell pluripotency or differentiation-associated genes by down-regulating Gadd45g. The expression of Nanog, Sox2 and Nestin, which were also observed to be regulated by miR-383, was unchanged by Gadd45g depletion. As is well known, each microRNA generally has hundreds of direct or indirect targets [6]. Since Nanog, Sox2 and Nestin are not potential targets predicted by the computer-aided algorithms (TargetScan, miRBase and Picta), miR-383 may indirectly regulate these genes through targets other than Gadd45g. We propose that miR-383 functions through multiple targets that synergize so as to regulate ES cell differentiation.

In conclusion, we demonstrated that miR-383 negatively regulates Gadd45g in the process of genotoxic stress-induced apoptotic events and ES cell differentiation. Recently, multiple miRNAs have been reported to be associated with genotoxic agents responsiveness, which suggests that these miRNAs play potential roles in cancer development [44]. Thus, a more detailed understanding about the functions of miRNA and their targets might lead to the development of new drugs for cancer treatment.

## Materials and Methods

### Cell culture and differentiation

Human breast cancer cell line MCF-7 and MDA-MB-231 (both these two cell lines come from ATCC) were cultured in Dulbecco's modified Eagle's medium (DMEM, Gibco) supplemented with 10% fetal bovine serum (FBS, Gibco), 100 U/mL penicillin, and 100 µg/mL streptomycin at 37°C in an atmosphere of 5% CO_2_. The mouse embryonic stem cell line R1 was cultivated with mouse embryonic fibroblast feeder cells in DMEM supplemented with 15% FBS, 2 mM L-glutamine (Gibco), 0.1 mM MEM nonessential amino acid (Gibco), 140 µM 2–mercaptoethanol (Sigma), 100 U/mL penicillin, 100 µg/mL streptomycin and 1000 U/mL leukemia inhibitory factor (LIF, Chemicon) on gelatin-coated plates. ES cells were trypsinized from the plates, suspended in ES medium and then left in the plates for 30 min to remove differentiated cells and feeder cells before they were recultured on a new plate. ES cell differentiation was induced by adding 1 µM retinoic acid (RA, Sigma) in the absence of LIF and without feeder cells. The embryoid body (EB) was formed by means of a hanging drop culture for 2 days and cultured without LIF for the indicated days.

### Vector construction and transfection

The human Gadd45g cDNA without the 3′-UTR was amplified from the plasmid pcDNA3.1-Gadd45g which was provided by Professor Qian Tao (The Chinese University of Hong Kong). After PCR amplification, Gadd45g cDNA without the 3′-UTR was cloned into the EcoRI and XhoI sites of a pcDNA3.1 vector. The 3′-UTR of Gadd45g was amplified from MCF-7 genomic DNA and cloned into the SpeI and SacI sites of a pMIR-REPORT luciferase vector. The pMIR-Gadd45g-mut construct was generated by site-specific mutagenesis, as shown in Fig. 1B, using primers containing mutant sites for PCR. MiR-383 mimic and control RNA were synthesized by GenePharma (Shanghai, China). Anti-miR-383 and anti-control were purchased from Ambion. Sixteen hours before transfection, MCF-7 or MDA-MB-231 cells were seeded onto culture plates and transfected with 200 nM of miR-383 mimic or 100 nM of anti-miR-383 using lipofectamine 2000 (Invitrogen) according to manufacturers' instructions. For R1 cell transfection, cells were cultured on gelatin-coated 6-well plates without feeder cells, then 200 nM of miR-383 mimic or 100 nM of Gadd45g siRNA were introduced using lipofectamine 2000. After transfection, cells were cultured with LIF or RA for 3 days.

### UV irradiation and cisplatin administration

The cells or transfected cells were irradiated with UVC (254 nm) using a UV lamp CL-1000 Ultraviolet Crosslinker (UVP, Upland, CA). Before UV irradiation, the culture media was removed, cells were irradiated at 20–60 J/m^2^, and fresh media was added to the plates immediately after irradiation. Cells were then cultured at 37°C for various periods. For cisplatin treatment, cells were grown to 80% confluency, and cisplatin (Sigma) was dissolved in DMSO and added to the medium at 25 µM (MCF-7 and MDA-MB-231) or 10 µM (R1) concentration for 24 h. The DMSO vehicle served as the control.

### Cell viability test and apoptosis assay

The 3-(4,5-dimethylthiazol-2-yl)-2,5-diphenyltetrazolium bromide (MTT) assay was performed to analyze cell viability. MCF-7 or MDA-MB-231 cells were seeded on 96-well plates at a density of 5×10^3^ per well before transfection. At 24 h post-transfection, cells were treated with UV or cisplatin. After 12 h (UV) or 24 h (cisplatin), 0.5 mg/mL MTT was added to the medium for 4 h. DMSO was added to the plates after the medium was removed, and the OD value at 490 nm was determined by a microplate reader (BIO-RAD). Annexin-V/PI Apoptosis Detection Kit (KeyGEN) was used to assess cell apoptosis. Simply, Annexin-V and PI was used to label early and late apoptotic cells, respectively. After staining, cells were analyzed by FACS Calibur flow cytometer (BD Biosciences).

### Luciferase assay

MCF-7 cells were seeded in a 24-well plate and transfected with either 100 nM of miR-383 mimic or control, 100 ng of pMIR-Gadd45g 3′-UTR or pMIR-Gadd45g-mut, and 10 ng of pRL vector, using lipofectamine 2000 according to manufacturer's protocol. Alternatively, 100 nM of anti-miR-383 or negative control were cotransfected with 100 ng of pMIR-Gadd45g 3′-UTR supplemented with 10 ng of pRL vector. Transfection of each construct was performed in triplicate. Two days post transfection, luciferase assays were performed using the dual-luciferase reporter system (VIGOROUS, China) according to the manufacturer's instructions. The Firefly luciferase reading was normalized to that of Renilla luciferase.

### RNA extraction and quantitative real-time PCR

Total RNA was extracted from MCF-7, MDA-MB-231 and R1 cells using TriZol reagent (Invitrogen), and quantified with Nanodrop Spectrophotometer (ThermoFisher). For cDNA reverse transcription, 1 ug RNA was converted by using M-MLV reverse transcriptase (Promega). Quantitative real-time PCR was performed using SYBR Green Master Mix (CWBIO, Beijing, China) on IQ5 Real-Time Detection System (BIO-RAD), and data was normalized to GAPDH expression. The mature miR-383 and U6 expression levels were detected using a modified stem-loop RT-PCR method [50]. For miR-383 and U6 reverse transcription, the specific miR-383 reverse transcription primer used was: 5′-GTCGTATCCAGTGCAGGGTCCGAGGTATTCGCACTGGATACGACagccac-3′; the specific U6 RT primer is 5′-AACGCTTCACGAATTTGCGT-3′. The real time PCR primer of mature miR-383 (forward: 5′-GTGCAGGGTCCGAGGT-3′, reverse: 5′-AGATCAGAAGGTGATTGTGGCT-3′), and U6 (forward: 5′- CTCGCTTCGGCAGCACA -3′, reverse: 5′- AACGCTTCACGAATTTGCGT -3′).

### Protein extraction and Western blotting

At 48 h post-transfection, cells were lysed using NP-40 lysis buffer (50 mM Tris, pH 7.5, 150 mM NaCl, 1.0% NP-40, 2 mM EDTA, 10 mM NaF, 1 mM Na_3_VO_4_ and 2 mM PMSF). Cell extracts were subjected to electrophoresis in 12% acrylamide gels and then transferred to PVDF membranes (Millipore). Antibodies against Gadd45g (Abcam, 1∶500), Nanog (Cell Signaling Technology, 1∶3000) and β-actin (Abmart, 1∶2000) were used. HRP-conjugated goat anti-mouse or goat anti-rabbit antibodies (Abmart, 1∶5000) were used as secondary antibodies.

### Statistical analysis

The results are presented as means ± standard deviation (SD). Statistical significance was determined using Student's *t*-test. *P*–value <0.05 was considered statistically significant.

## Supporting Information

Figure S1 **Nuclear morphology and percentage of apoptotic cells showing condensed chromatin or apoptotic bodies at indicated time points (A) and dose (B) post UV irradiation.** Nuclei were stained with Hoechst 33258.
(TIF)

Figure S2 **Analysis by light microscopy revealed that overexpression of miR-383 increased the sensitivity to UV irradiation or cisplatin in MCF-7 (A) and MDA-MB-231 cells (B).**
(TIF)

Figure S3 (A) MDA-MB-231 cells were transfected with miR-383 mimic or control, and treated with UV irradiation (60 J/m2, post 12 h) or cisplatin (25 µM, post 24 h). MTT assays were performed as indicated in materials and methods. (B) Apoptosis was analyzed by Annexin V/PI assay.
(TIF)
