# Supplementary figures and images for: MicroRNA-383 Regulates the Apoptosis of Tumor Cells through Targeting Gadd45g
Source: PLoS One. 2014 Nov 21;9(11):e110472. doi: 10.1371/journal.pone.0110472 (PMC4240536; doi:10.1371/journal.pone.0110472)

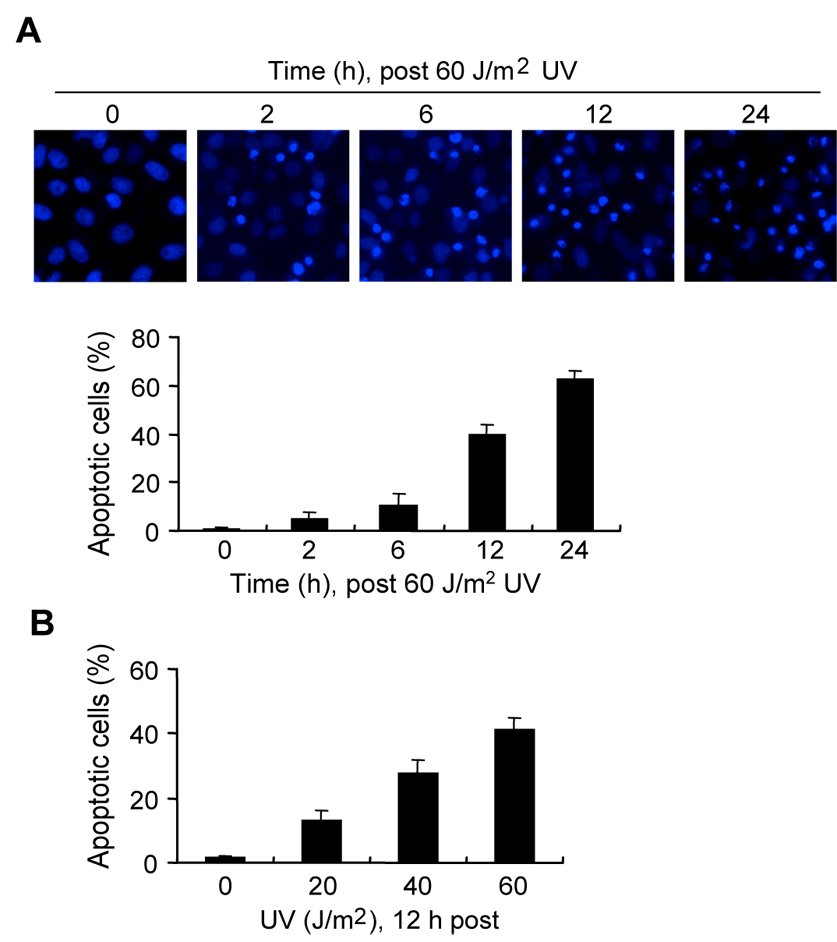

Supplement: Figure S1 — Nuclear morphology and percentage of apoptotic cells showing condensed chromatin or apoptotic bodies at indicated time points (A) and dose (B) post UV irradiation. Nuclei were stained with Hoechst 33258. (TIF) [file pone.0110472.s001.tif]

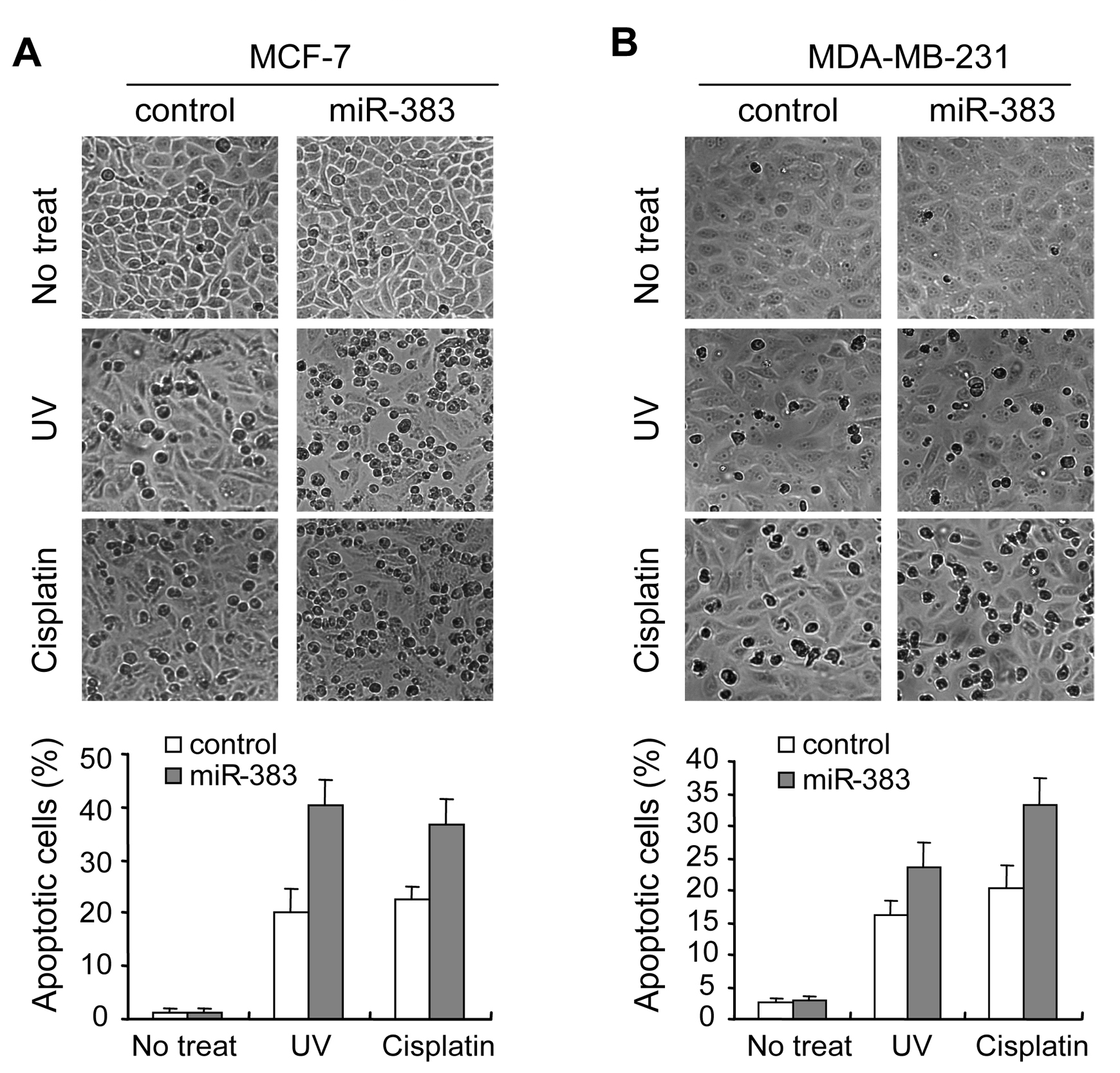

Supplement: Figure S2 — Analysis by light microscopy revealed that overexpression of miR-383 increased the sensitivity to UV irradiation or cisplatin in MCF-7 (A) and MDA-MB-231 cells (B). (TIF) [file pone.0110472.s002.tif]

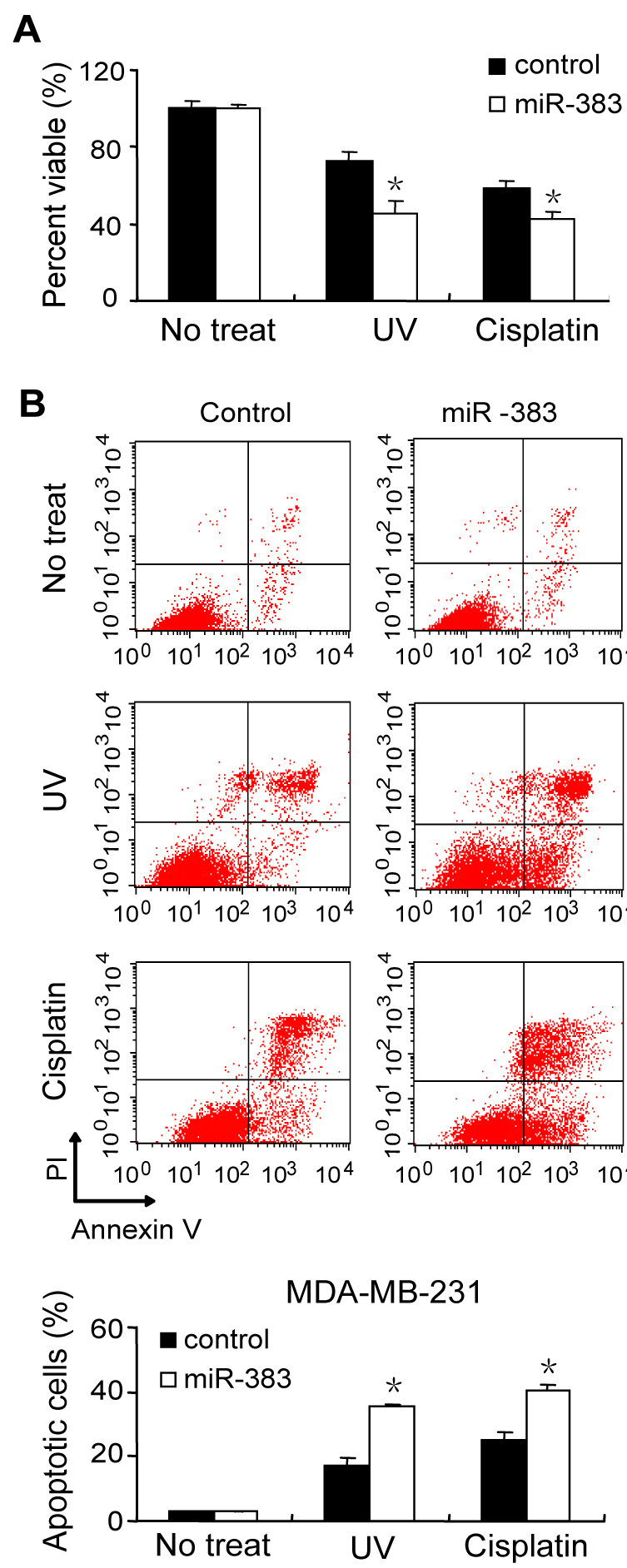

Supplement: Figure S3 — (A) MDA-MB-231 cells were transfected with miR-383 mimic or control, and treated with UV irradiation (60 J/m2, post 12 h) or cisplatin (25 µM, post 24 h). MTT assays were performed as indicated in materials and methods. (B) Apoptosis was analyzed by Annexin V/PI assay. (TIF) [file pone.0110472.s003.tif]
